# Novel Therapeutic Approaches Enhance PGC1-alpha to Reduce Oxidant Stress-Inflammatory Signaling and Improve Functional Recovery in Hibernating Myocardium

**DOI:** 10.3390/antiox11112155

**Published:** 2022-10-31

**Authors:** Rishav Aggarwal, Koray N. Potel, Edward O. McFalls, Tammy A. Butterick, Rosemary F. Kelly

**Affiliations:** 1 Division of Cardiothoracic Surgery, Department of Surgery, University of Minnesota Medical School, Minneapolis, MN 55455, USA; 2 School of Medicine, Dentistry and Biomedical Sciences, Queen’s University Belfast, Belfast BT9 7BL, UK; 3 Division of Cardiology, Richmond VA Medical Center, Richmond, VA 23249-4915, USA; 4 Department of Neuroscience, University of Minnesota, Minneapolis, MN 55455, USA; 5 Department of Research, Center for Veterans Research and Education, Minneapolis, MN 55417, USA

**Keywords:** PGC1-alpha, oxidative stress, NF-κB, hibernating myocardium, mitochondrial metabolism, mesenchymal stem cell

## Abstract

Ischemic heart disease affects millions of people around the world. Current treatment options, including coronary artery bypass grafting, do not result in full functional recovery, highlighting the need for novel adjunctive therapeutic approaches. Hibernation describes the myocardial response to prolonged ischemia and involves a set of complex cytoprotective metabolic and functional adaptations. PGC1-alpha, a key regulator of mitochondrial energy metabolism and inhibitor of oxidant-stress-inflammatory signaling, is known to be downregulated in hibernating myocardium. PGC1-alpha is a critical component of cellular stress responses and links cellular metabolism with inflammation in the ischemic heart. While beneficial in the acute setting, a chronic state of hibernation can be associated with self-perpetuating oxidant stress-inflammatory signaling which leads to tissue injury. It is likely that incomplete functional recovery following revascularization of chronically ischemic myocardium is due to persistence of metabolic changes as well as prooxidant and proinflammatory signaling. Enhancement of PGC1-alpha signaling has been proposed as a possible way to improve functional recovery in patients with ischemic heart disease. Adjunctive mesenchymal stem cell therapy has been shown to induce PGC1-alpha signaling in hibernating myocardium and could help improve clinical outcomes for patients undergoing bypass surgery.

## 1. Introduction

Ischemic Heart Disease (IHD) affects well over one hundred million patients worldwide and remains one of the leading causes of death. Although advances in medical management have led to a decrease in age-adjusted rates, the prevalence of IHD continuously rises, as population ageing increases the overall disease burden [1]. Coronary artery bypass grafting (CABG) aims to surgically restore blood flow and oxygen supply to ischemic heart tissue [2]. Studies have shown, however, that the current medical and surgical management of IHD, including CABG, does not lead to full functional recovery of chronically ischemic myocardium [3,4]. Therefore, additional therapeutic options are needed to ensure optimal long-term outcomes for patients suffering from IHD. In recent years, studies have looked at the metabolic changes and functional adjustments seen in ischemic heart tissue and have improved our understanding of the adaptive process called hibernation that is observed in chronically hypoxic myocardium that is viable but with reduced function. Like in other cardiovascular diseases, mitochondrial metabolism, oxidative stress and inflammatory signaling seem to play a central role in myocardial dysfunction and recovery [5]. Novel therapies, therefore, aim to expand treatment approaches and improve functional outcomes in IHD patients by targeting key signaling pathways involved in cell metabolism and mitochondrial biogenesis. 

## 2. Hibernating Myocardium and Revascularization

**Figure 1:**
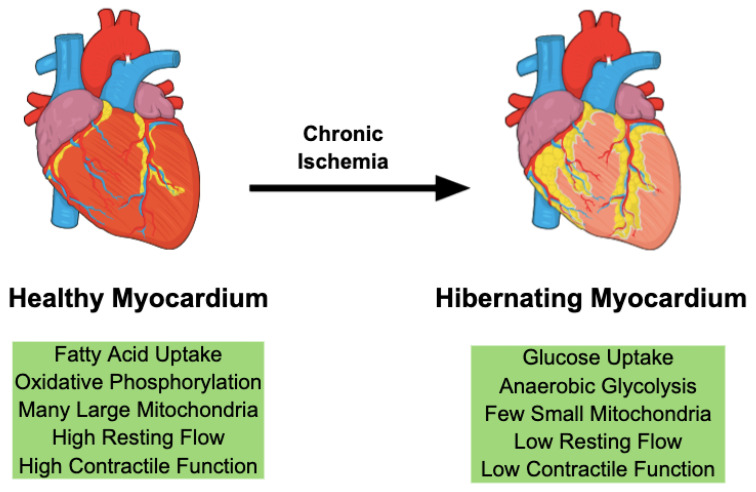
Chronic ischemia initiates hibernation in cardiac myocytes. This adaptive response involves a coordinated change in gene expression leading to increased glucose uptake and glycolysis-dependent metabolism. On histology, hibernating myocardium is characterized by fewer and smaller mitochondria.

Hibernation describes the multifaceted phenotypic adaptations in chronically ischemic myocardium (Figure 1). Reduced blood supply secondary to coronary artery stenosis leads to chronic regional hypoxia and triggers adaptive mechanisms that try to reestablish an equilibrium between oxygen demand and supply. Reducing the metabolic activity to a level that matches oxygen availability prevents irreversible cardiomyocyte injury and infarction [6]. Our studies support the notion that these metabolic changes protect the myocardium from harmful ischemia by allowing cardiomyocytes to operate at a lower level of the oxygen supply-demand relationship [7]. These adaptations preserve myocardial viability at the expense of contractile function, leading to a phenotype characterized by decreased regional blood flow and cardiac function in the absence of tissue necrosis [8,9]. 

Unlike in myocardial infarction, hibernation allows cardiomyocytes to regain their functionality if the oxygen supply is restored. To preserve cell viability, cardiomyocytes deploy a complex program of gene expression changes, which increases cardioprotective gene expression and simultaneously downregulates metabolic proteins [10,11]. Over time, metabolic indices in ischemic myocardium improve, as these adaptations reduce the mismatch of oxygen supply and metabolic demands [12]. The proteomic profile of hibernating myocardium is characterized by an upregulation of stress and cytoskeletal proteins as well as downregulation of mitochondrial and contractile proteins [13,14]. Cells shift their metabolism to reduce the dependency on oxygen availability by increasing glucose uptake and anaerobic metabolism while decreasing their oxidative mitochondrial activity, leading to abnormally small mitochondria on histology [15]. Signs of active inflammation are found in hibernating myocardial tissue [16]. Proinflammatory factors can contribute to cardio-protection by regulating myocardial function and cell survival [17]. While these adaptive mechanisms preserve tissue viability in the acute setting, compensations are often insufficient in the long term and can eventually lead to chronic dysfunction and injury [18]. 

Surgical treatment of chronically ischemic myocardium tries to reestablish sufficient blood supply to allow the cardiac tissue to return to its previous metabolic and functional state. CABG restores the oxygen supply to hibernating regions and reverses many of the adaptive mechanisms triggered by chronic ischemia. We have shown, however, that hibernating myocardium does not fully recover after successful revascularization and continues to exhibit reduced function and flow when challenged with dobutamine. Our molecular analyses show that mitochondrial proteins involved in oxidative metabolism continue to be reduced in revascularized hibernating myocardium, which could be a possible cause of the incomplete functional recovery [19,20]. To offer patients the best long-term outcome by fully restoring cardiac function to pre-ischemic levels, clinicians and researchers need to consider targeting the remaining metabolic deficits in revascularized myocardium using novel adjunctive therapies. In recent years, studies have uncovered the important role the peroxisome proliferator-activated receptor-gamma coactivator (PGC)1-alpha plays in mitochondrial metabolism as well as its regulatory effects on oxidative stress and inflammation. PGC1-alpha might therefore be a promising target for future therapies that try to overcome current limitations in the treatment of chronically ischemic myocardium.

## 3. PGC1-alpha Is a Key Mediator of Mitochondrial Energy Metabolism

Myocardium has a high metabolic demand and derives its energy in the form of adenosine triphosphate (ATP) by oxidating fatty acids and glucose. In a normally functioning adult heart, 98% of ATP is generated through the process of oxidative phosphorylation inside the mitochondrial membrane. Only about 2% of ATP is derived from anaerobic glycolysis within the cytoplasm [21]. The vast majority of energy is generated by oxidation of fatty acids [22]. The delicate balance between fatty acid- and glucose-dependent metabolism is controlled by a multitude of factors and can be disrupted in various heart diseases [23]. Since cardiomyocytes have a low division rate and a long cellular lifespan, control mechanisms need to ensure the ongoing functional capacity of mitochondria. This is achieved through mitochondrial quality control mechanisms, which are responsible for the maintenance, removal and biogenesis of mitochondria [24]. 

The transcriptional coactivator PGC1-alpha has been shown to be a key mediator in mitochondrial energy metabolism, by regulating fatty acid and glucose oxidation, as well as controlling key components of mitochondrial quality control [22,23]. It belongs to the PGC1 family of transcriptional coactivators, alongside PGC1-beta and PGC1-related coactivator. PGC1-alpha is highly expressed in tissues with a high oxidative capacity, including myocardium, brown adipose tissue, and skeletal muscle [5]. Through coactivation and significant potentiation of transcription factor activity, PGC1-alpha shifts cellular metabolism towards increased oxidative phosphorylation. It amplifies the uptake and utilization of fatty acids for oxidative metabolism and plays a key role in ensuring mitochondrial quality control. It regulates gene expression through binding to DNA transcription factors and the formation of transcription-initiating enzymatic protein complexes [25,26,27]. Using these coactivating mechanisms, PGC1-alpha has been shown to regulate multiple metabolic pathways both inside and outside of mitochondria and is a key regulator of intracellular responses to physiological stimuli, such as hypothermia, hypoxia and starvation [25,28,29]. This allows cells to adjust mitochondrial biogenesis and energy metabolism to extracellular conditions and demands [30]. Importantly, PGC1-alpha increases mitochondrial respiratory capacity, enhances ATP production through oxidation of fatty acids and induces mitochondrial biogenesis, resulting in an overall increased mitochondrial mass [5,22,28]. In addition, its activation facilitates the transcription of nuclear genes that control mitochondrial repair and removal [22].

**Figure 2:**
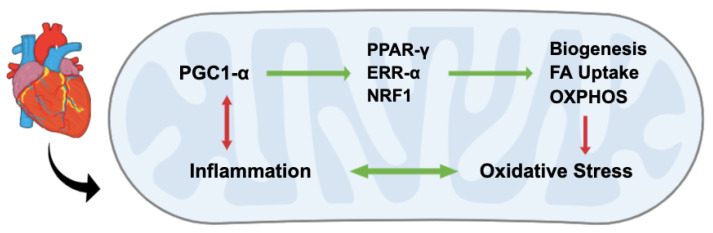
PGC1-alpha is a key mediator of mitochondrial metabolism. Through coactivation of transcription factors, including PPAR-gamma, EER-alpha and NRF1, PGC1-alpha enhances mitochondrial biogenesis, uptake of fatty acids (FA) and oxidative phosphorylation (OXPHOS). It suppresses NF-κB-mediated inflammation and oxidative stress. Importantly, proinflammatory signaling downregulates PGC1-alpha, resulting in decreased oxidative metabolism and increased oxidative stress.

Key transcription factors that are coactivated by PGC1-alpha include peroxisome proliferator-activated receptor (PPAR)-alpha, Nuclear Respiratory Factor 1 (NRF1) and Estrogen-related receptor (EER)-alpha [5,30,31]. In myocardium, PPAR-alpha has been shown to promote uptake and utilization of fatty acids for ATP production. PGC1-alpha coactivates PPAR-alpha and promotes its action through the formation of the PPAR-alpha-PGC1-alpha protein complex [27]. NRF1 and EER-alpha both play key roles in mediating mitochondrial biogenesis and oxidative metabolism [23,32]. Alongside NRF2, these transcription mediators are activated by PGC1-alpha and not only increase mitochondrial protein synthesis but also have numerous effects on oxidative stress and inflammation by reducing reactive oxygen species (ROS), stimulating the release of antioxidants, and suppressing the proinflammatory nuclear factor kappa B (NF-κB) signaling pathway (Figure 2) [26,33,34].

PGC1-alpha has been shown to be highly expressed in cardiomyocytes [35]. Its up- and downregulation results in a series of metabolic changes that are implicated in both physiologic and pathologic cardiac conditions. In the healthy adult heart, PGC1-alpha expression ensures sufficient uptake and oxidation of fatty acids for ATP production [23]. Stressors that increase myocardial energy demand are known to upregulate PGC1-alpha signaling. For example, physical exercise increases PGC1-alpha leading to a so-called “athlete’s heart” phenotype, which is characterized by increased biogenesis and oxidative metabolism—adaptations that increase energy availability in situations of increased demand [36]. The effects of exercise on PGC1-alpha expression have also been studied in other tissues, including striated muscle. Contractile activity seems to induce PGC1-alpha and associated mediators including NRF1 while decreasing the release of key inflammatory cytokines, including TNF-alpha and IL-6 [35,37]. Downregulation of PGC1-alpha, on the other hand, can be observed in a context of decreased energy demand or oxygen availability. Denervation of striated muscle has been shown to a reduction in PGC1-alpha signaling and an increase in oxidative stress and apoptosis [38]. Ischemia is also known to trigger a downregulation of PGC1-alpha. In response to decreased blood flow and oxygen availability, cardiomyocytes decrease PGC1-alpha signaling to reduce ATP production through oxygen-dependent oxidative phosphorylation. This results in increased anaerobic glycolysis, decreased oxidative fatty acid metabolism and context-dependent cardiac contractile dysfunction [21]. Hence, PGC1-alpha signaling appears to play a crucial role in the myocardial response to stressors, such as exercise and ischemia [39]. In certain contexts, however, it seems difficult to distinguish between physiological adaptations in response to altered energy demands and pathological dysregulation of PGC1-alpha associated signaling. In heart failure, for example, PGC1-alpha signaling has been shown to be disrupted, without clear evidence whether these changes preserve or further disrupt cardiac function and viability [23,40].

In vitro cultures and animal models have been used to further study the effects of PGC1-alpha overexpression and knock-out on myocardial development and function [41]. Overexpression of PGC1-alpha leads to uncontrolled mitochondrial biogenesis in cultured cardiomyocytes [28]. In mouse models, the effects of overexpression crucially depended on the animal’s developmental stage and baseline metabolism. In adult wild type mice, PGC1-alpha overexpression resulted in cardiac degeneration, development of cardiomyopathy and an overall reduced lifespan [28,42]. In a model of conditional PGC1-alpha enhancement, the resulting hypertrophy and ventricular dysfunction could be fully reversed upon cessation of overexpression [43]. Interestingly, PGC1-alpha enhancement was also shown to increase levels of acylcarnitines—an ischemic metabolite and marker of tissue damage [44]. Not only have acylcarnitines known cardiotoxic effects by disrupting both calcium homeostasis and mitochondrial energy metabolism but were also shown to induce NF-κB [45,46]. Paradoxiacally, the PGC1-alpha mediated increase in acylcarnitines, which is likely driven by an imbalance of oxidation and lipid uptake, was reversed by exercise [44,47]. In mice with reduced baseline PGC1-alpha levels, signaling enhancement had beneficial effect on cell metabolism and survival [42]. Similarly, in the fetal heart, which is known to express lower levels of PGC1-alpha and only increases its oxidative metabolism in response to increased physiologic strain following birth, signaling enhancement increased biogenesis and oxidative metabolism without causing pathologic remodeling [23,28,43]. PGC1-alpha knock-out, on the other hand, resulted in a reduction in mitochondrial proteins in mouse models [48]. Germline and postnatal knock-out led to profound alterations in mitochondrial morphology and function, which blunted cardiac muscle growth and caused severe cardiomyopathies [41,49]. In adult mice, decreased PGC1-alpha signaling blunted enzymatic activity [50]. Mice lacking PGC1-alpha also developed severe cardiac dysfunction when challenged by fluid overload [39,51]. The observation that contractile dysfunction due to PGC1-alpha deficiency can be very subtle under normal conditions but becomes obvious under increased strain could suggest that PGC1-alpha signaling allows rapid adaptations in situations of increased workload and is crucial in contexts that require increased energy consumption [50]. 

In summary, PGC1-alpha is highly expressed in myocardium and plays a central role in energy metabolism as well as mitochondrial biogenesis, while regulating both oxidative stress and inflammatory signaling. 

## 4. PGC1-alpha Regulates Oxidative Stress and Biogenesis

**Figure 3:**
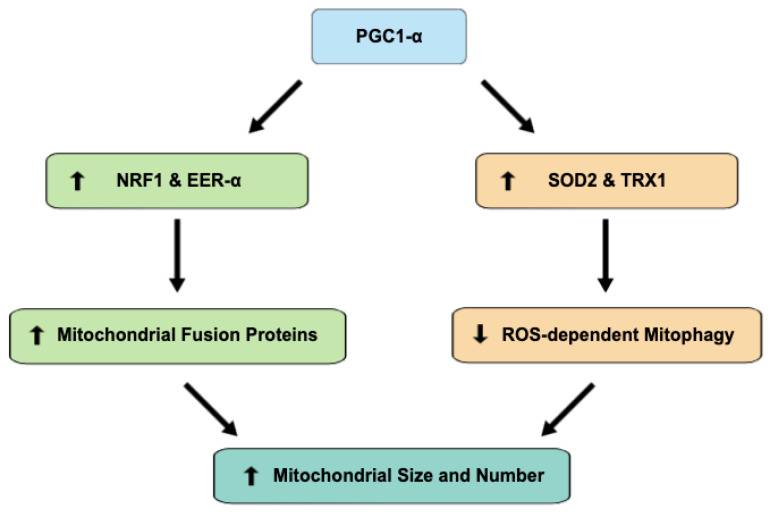
PGC1-alpha signaling increases mitochondrial size and number through means of both enhanced biogenesis and decreased mitophagy. Co-activation of transcription factors NRF1 and EER-alpha increases the expression of mitochondrial fusion proteins, including mitofusins-1 and 2, while the antioxidant effects of SOD2 and TRX1 co-activation decrease ROS-dependent mitophagy.

ROS are a byproduct of mitochondrial respiration. Enhanced mitochondrial metabolism will therefore increase harmful cellular oxidative stress in the absence of sufficient detoxifying mechanisms. PGC1-alpha induces antioxidant enzymes, such as SOD2 and TRX1, to counteract increased ROS generation secondary to mitochondrial respiration enhancement. PGC1-alpha signaling is known to be induced in response to oxidative stress, leading to increased antioxidant release and allowing the cell to adapt to prooxidant stressors. While decreased PGC1-alpha makes cells more susceptible to the harmful effects of oxidative stress, increased levels have been shown to serve a protective function [52,53]. PGC1-alpha knockout in a mouse model resulted in a reduction in antioxidant enzymes, including SOD2, whereas PGC1-alpha enhancement using a PPAR-gamma or AMPK agonist raised SOD2 levels [54,55]. Further studies revealed that a reduction in PGC1-alpha signaling results in ROS-dependent mitophagy, while its induction promotes mitochondrial growth [56]. Similarly, we observed higher levels of PGC1-alpha to be associated with increased expression of mitofusins-1 and 2, which play a key role in mitochondrial fusion and serve as indicators of mitochondrial biogenesis [57,58]. Hence, by enhancing biogenesis and inhibiting ROS-dependent mitophagy, protective PGC1-alpha signaling links mitochondrial activity with detoxifying mechanisms and regulates the cellular metabolic balance (Figure 3) [56,59].

Through enhancing antioxidant enzyme expression, PGC1-alpha is a key regulator of the myocardial response to stressors. While PGC1-alpha knockout in murine model did not result in significantly increased oxidative stress at baseline, mice developed cardiac hypertrophy and contractile dysfunction under pressure overload. Hypertrophy and dysfunction were attenuated by administration of an antioxidant enzyme mimetic, highlighting how the regulation of antioxidant responses by PGC1-alpha plays a crucial protective role in situations of increased cellular stress [54].

The FoxO family of transcription factors is coactivated by PGC1-alpha and has been shown to be of importance for cardiac development and stress response. PGC1-alpha coactivation allows FoxO transcription factors to facilitate the expression of antioxidant enzymes and key cardiac proteins, including ion channels. Disruptions in PGC1-alpha signaling significantly impairs FoxO activity which results in decreased ROS detoxification and cardiac dysfunction [60,61]. Intriguingly, the detrimental effects of PGC1-alpha/FoxO signaling disruption become significant in situations of increased physiological stress, such as pressure overload [62]. These observations highlight that PGC1-alpha is a critical component of cellular antioxidant mechanisms and support the notion that disruption of this signaling pathway significantly impairs the myocardial ability to sufficiently adapt to external stressors, such as ischemia or fluid overload as seen in IHD and heart failure.

## 5. PGC1-alpha Links Cellular Metabolism and Inflammation in the Ischemic Heart

PGC1-alpha signaling has emerged as one of the central regulators of mitochondrial metabolism, oxidative stress and inflammation. Through the coactivation of transcription factors, PGC1-alpha increases oxidative fatty acid metabolism and induces mitochondrial biogenesis, facilitating maximum cardiac function in the healthy heart. In myocardial ischemia, however, when decreased oxygen availability limits mitochondrial oxidative capacity, cardiomyocytes adjust their energy production by increasing glucose uptake and anaerobic metabolism in an effort to reduce their dependency on oxidative phosphorylation. Consequently, hibernating myocardium has been shown to express lower levels of PGC1-alpha [18,63]. 

An increase in ROS and inflammatory cytokines is observed in response to hypoxia and anaerobic metabolism in hibernating myocardium. Ischemic stress induces cytokine production which in turn promotes local inflammation and regulates cell survival through NF-κB activation [17,64,65]. Activation of the p38 mitogen-activated protein kinase (MAPK) signaling pathway is enhanced in hibernating myocardium, which increases glucose uptake, induces proinflammatory cytokines, and activates both NF-κB and inducible nitric oxide synthase (iNOS) [65,66,67]. The release of cytokines, including TNF-alpha, stimulation of iNOS and activation of NF-κB form part of a coordinated cardioprotective response in the early stages of myocardial ischemia and hibernation. These proinflammatory mediators have been shown to regulate cell survival and limit cardiac contractility due to their negative inotropic effects. Reducing cardiac contractility prevents metabolic demands from drastically exceeding mitochondrial energy metabolism, thereby limiting hypoxic cell injury. Proinflammatory and prooxidant signaling, therefore, contributes to the cardioprotective mechanisms seen in hibernating myocardium. However, importantly, chronic activation of these pathways can lead to myocardial injury, pathologic remodeling and cell death [17,68]. ROS have been shown to cause cell injury, while proinflammatory cytokines, such as TNF-alpha, IL-1 and IL-6, appear to play a central role in the pathological mechanisms underlying the development of heart failure [18,69]. It is therefore likely, that proinflammatory and prooxidant signaling pathways contribute to cardiac protection in the early stages of hypoxia and hibernation but cause myocardial injury and chronic dysfunction in the context of prolonged upregulation. Chronic activation of these pathways is facilitated by their reciprocal stimulation which results in a self-perpetuating signaling cascade. While ROS promote inflammation through p38 MAPK activation, proinflammatory cytokines are known to increase ROS production [17,67]. This perpetual, reciprocal activation can lead to a self-sustaining proinflammatory and prooxidant environment, which causes irreversible damage and dysfunction in the absence of regulatory factors. 

PGC1-alpha is known to be a key regulator of oxidative stress and inflammation. It stimulates the release of antioxidant mediators and suppresses NF-κB signaling [26,33,70]. However, the induction of NF-κB in ischemic myocardium has been shown to actively downregulate PGC1-alpha expression, resulting in chronic suppression of PGC1-alpha and further disinhibition of NF-κB signaling [63]. Furthermore, proinflammatory cytokines, such as TNF-alpha, decrease PGC1-alpha expression via NF-κB and p39 MAPK pathways [71]. This mutual suppression results in a regulatory antagonism between PGC1-alpha-induced oxidative metabolism and oxidant stress-inflammatory signaling [72]. In the context of myocardial ischemia and hibernation, prooxidant and proinflammatory mediators can therefore not only control cell survival and contractility but also enhance glucose uptake and anaerobic metabolism by suppressing PGC1-alpha signaling [73].

Considering these mechanisms of metabolic and inflammatory signaling in hibernating cardiomyocytes, PGC1-alpha emerges as a key regulator and promising target of novel therapies aiming to overcome the incomplete recovery of revascularized myocardium. Not only is PGC1-alpha an important mediator in healthy heart tissue, but its activity also appears able to reverse the numerous metabolic and inflammatory adaptations observed in hibernating myocardium. Enhancement of PGC1-alpha signaling in revascularized myocardium might therefore prevent ongoing oxidative stress and inflammation while facilitating full recovery of mitochondrial metabolism. Allowing cell metabolism to return to its pre-ischemic state would likely improve contractility and increase the maximal functional capacity. Researchers, therefore, investigate the potential benefits of PGC1-alpha enhancing therapies in the context of hibernating myocardium. 

## 6. Enhanced PGC1-alpha Signaling Improves Recovery in Hibernating Myocardium 

Recent studies investigated the potential of PGC1-alpha enhancement using various interventions, including dietary supplements, drugs and stem cell therapies (Table 1). Based on in vitro and in vivo studies of PGC1-alpha modification in cardiomyocytes, normalizing and fine tuning previously disrupted signaling might add cardioprotective benefits to current treatment approaches [42]. Pioglitazone, a PPAR-gamma receptor agonist, increased PGC1-alpha and mitochondrial antioxidant protein expression in a porcine model of hibernating myocardium [74]. Using a similar model, administration of dietary CoQ10, a key component of the electron transport chain (ETC), has been shown to increase the expression of PGC1-alpha, enhance mitochondrial biogenesis, and reduce NT-pro BNP levels, a marker of myocardial injury [75,76,77]. These results suggest that noninvasive therapies could be used to enhance PGC1-alpha signaling and might reduce cell injury in hibernating myocardium.

**Figure 4:**
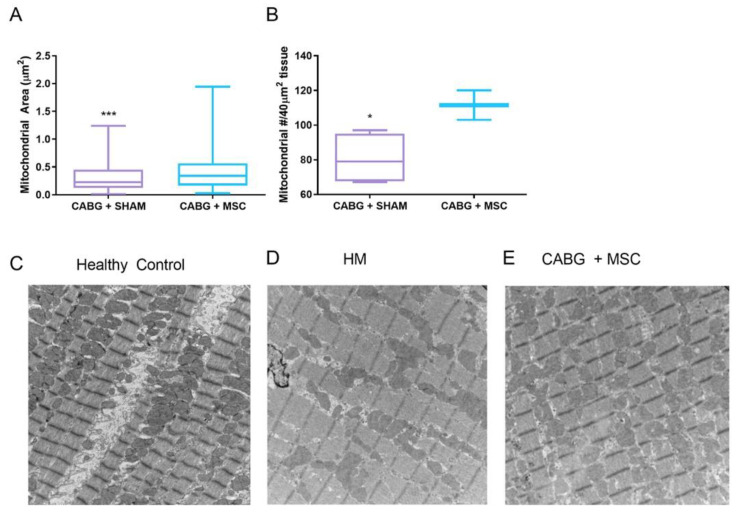
Dysregulation of mitochondrial size and number can be observed in hibernating myocardium. Mesenchymal stem cells have shown to increase mitochondrial size and density when used alongside CABG (**A**,**B**). Comparing healthy and hibernating myocardium (HM), electron microscopy demonstrates mitochondrial dysregulation associated with hibernation (**C**,**D**) and significant recovery following CABG and stem cell treatment (**E**). * *p* < 0.05; *** *p* < 0.001

Other studies tried to investigate the effects of mesenchymal stem cell (MSC) therapy on hibernating myocardium. Mitochondrial function and ATP production has been shown to increase in hypoxic cardiomyocytes when co-cultured with MSCs upon reoxygenation. The likely mechanism driving this metabolic shift is increased PGC1-alpha expression stimulated by paracrine MSC signaling [80]. Similarly, MSCs appear to preserve PGC1-alpha levels when transplanted into infarcted myocardium, resulting in an increased energetic state and improved cardiac function [81]. In our swine model, the application of an MSC cardiac patch onto hibernating myocardial regions during CABG enhanced the expression of PGC1-alpha and ETC proteins. Intriguingly, further histological and functional analyses showed that MSC therapy not only increased mitochondrial size and density but also led to a significant improvement in cardiac function (Figure 4) [79].

Since overexpression of PGC1-alpha has been shown to have beneficial effects on cellular metabolism and function, these novel treatment approaches could provide promising adjunctive therapies for patients suffering from IHD [82]. By promoting the reversal of the hibernation phenotype in revascularized myocardium, restoring mitochondrial oxidative capacity and breaking the self-perpetuating cascade of prooxidant and proinflammatory signaling, PGC1-alpha enhancing therapies could overcome the shortcomings of current management options and improve long-term outcomes for IHD patients.

## 7. Conclusions

Hibernation describes the myocardial adaptations in response to chronic ischemia. As opposed to infarcted heart tissue, hibernating myocardium continues to be viable and is characterized by downregulation of oxidative metabolism and mitochondrial biogenesis, a low resting flow and contractile dysfunction. Surgical revascularization can restore sufficient blood flow to previously ischemic myocardium, however, studies showed incomplete functional recovery of hibernating myocardium following revascularization. The remaining functional impairment might be due to persisting metabolic adaptations, which do not fully resolve after sufficient oxygen supply is restored and might prevent full recovery.

PGC1-alpha is a key mediator of mitochondrial metabolism and is highly expressed in cardiomyocytes. It enhances oxidative phosphorylation and upregulates key genes involved in mitochondrial quality control. PGC1-alpha signaling is thought to play a key role in the myocardial stress response and allows metabolic adaptations in situations of increased energy demand and reduced oxygen supply. Importantly, PGC1-alpha signaling suppresses oxidative stress and proinflammatory signaling by enhancing antioxidant expression and downregulating NF-κB. Both prooxidant and proinflammatory signaling are known to be increased in hibernating myocardium, while PGC1-alpha expression is suppressed. Following surgical revascularization, PGC1-alpha continues to be decreased in previously hibernating myocardium which could be the reason for the remaining contractile dysfunction, especially under increased strain. Novel PGC1-alpha enhancing interventions, including drugs and mesenchymal stem cells, could be used as adjunctive therapies and further improve long-term outcomes for patients with IHD by fully reversing the hibernation phenotype and restoring optimal cellular metabolism in chronically ischemic myocardium.

## Figures and Tables

**Table 1:** Overview of in vivo studies investigating PGC1-alpha enhancing interventions.

Type	Intervention	Model	Sample	Biopsied Tissue	Reference
Lifestyle-based	Exercise	Human	n = 7	Skeletal Muscle	[78]
Dietary	CoQ10	Swine	n = 12	Myocardium	[75]
Pharmacological	Pioglitazone	Swine	n = 18	Myocardium	[74]
Cell-therapeutic	Mesenchymal Stem Cells	Swine	n = 12	Myocardium	[79]

Note: All studies found significantly increased PGC1-alpha expression with *p*-values < 0.05.
